# Management of cognitive decline in Alzheimer's disease using a non-pharmacological intervention program: A case report

**DOI:** 10.1097/MD.0000000000020128

**Published:** 2020-05-22

**Authors:** Zara Quail, Mark Mclean Carter, Angelina Wei, Xinlei Li

**Affiliations:** a Care Visions Limited, Stirling, Scotland, UK; b Care Visions China, Beijing, China.

**Keywords:** Alzheimer's disease, dementia, non-pharmacological interventions, social care, social participation

## Abstract

**Introduction:**

In China, the over 60 population is estimated to grow from 12% in 2010 to 33% of the overall population by 2050. The escalation in the aging population is projected to result in an Alzheimer's disease prevalence of 27.7 million people in China by 2050 causing substantial health and economic burden. While there are some published studies on multicomponent, non-pharmacological interventions for people with dementia, we have found no published community-based approach to care that encompasses personalized selection of non-pharmacological interventions, active social participation, and dementia education.

**Patient concerns:**

An elderly female living at home alone in urban Beijing presented with significant short-term memory impairment, episodes of confusion, difficulty with language skills, and episodes of wandering. She had become reclusive and disengaged from her previous social networks, and no longer attended any community activities or events. The patient had no significant past medical or psychiatric history.

**Diagnosis:**

The patient was diagnosed with Alzheimer's disease by a local physician based on clinical features of impaired communication, disorientation, confusion, poor judgement, behavioral changes, and difficulty speaking. Depression was considered a differential diagnosis but is also both a risk factor and symptom of dementia.

**Interventions:**

A novel, community-based, multicomponent social care program for dementia was used to facilitate implementation of non-pharmacological interventions, gradual socialization and provide supportive carer and community education. Non-pharmacological interventions included a combination of validation therapy, music therapy, art therapy, reminiscence therapy, talking therapy, reality orientation, cognitive training, smell therapy, food therapy, sensory stimulation, garden therapy, and physiotherapy.

**Outcomes:**

Improvements in the patient's Geriatric Depression Scale and Mini Mental State Examination scores were noted in association with increased social participation in the community.

**Conclusion:**

The community-based, multicomponent dementia social care program described in this case report has enabled a socially isolated patient with Alzheimer's disease to reduce her social isolation with an associated improvement in her mood and prevention of cognitive decline. Educating the community was an essential part of re-integrating the patient into the social setting. Reducing social isolation and increasing community engagement were essential to maintaining the patient's independence in her own home.

## 1 Introduction

In China the population over the age of 60, in which Alzheimer's disease becomes more prevalent, is estimated to grow from 12% in 2010 to 33% of the overall population by 2050, resulting in a projected Alzheimer's disease prevalence of 27.7 million people in China by 2050.^[1]^ Consequently, there will be a substantial increase in the health and economic burden of Alzheimer's disease on care givers, health care facilities, health care providers, and communities.^[1]^ In China, the challenge of increasing dementia prevalence is compounded by barriers to dementia diagnosis and care including the stigma of dementia, lack of medical school training and resultant lack of health care professionals confident in dementia assessment and management, lack of access to diagnostic blood tests and imaging, and few memory clinics.^[2–5]^ Culturally in China, there is a priority of wanting to live at home for as long as possible, but changing socioeconomic environments mean that family members may not be able to maintain home-based care, especially for those who have to migrate for work.^[6]^ As a consequence, there is a requirement for interventions and community-based supports for people with dementia to help maintain independence within the community and at home.^[7]^ While there is no pharmacological cure for dementia, there is a growing body of evidence for individual and multicomponent non-pharmacological interventions showing benefits for cognition, neuropsychiatric symptoms, daily functioning, and quality of life.^[8–19]^ While there are also some published studies on multicomponent, non-pharmacological interventions for people with dementia that combine activities for the physical, intellectual, functional, and social stimulation,^[14–19]^ we have found no published community-based approach to guide care that encompasses personalized selection of non-pharmacological interventions, active social participation, and dementia education.

This case report describes an elderly female with features of cognitive decline and depression, living at home alone, who was referred to our social care service after diagnosis of Alzheimer's disease. Depression is a risk factor for, and common neuropsychiatric symptom of, dementia.^[20]^ Both dementia and depression can result in cognitive and functional impairment and deterioration in quality of life.^[20]^ Social isolation is a both risk factor for, as well as potential consequence of dementia and depression.^[20]^ With high levels of undetected dementia and depression in older people in China,^[21]^ where specialized dementia social care services remain rare,^[2]^ there is a need for practical, symptom-focused solutions to delivering dementia care in the community. Our dementia social care delivery team has developed a novel, community-based, multicomponent, non-pharmacological intervention program for the management of people with Alzheimer's disease and dementia.

## 2 Case report

Patient information: A widowed, retired chemistry teacher in her late 70s was living alone in a 2-bedroom apartment in urban Beijing, China. In 2013, her children noticed significant short-term memory impairment, episodes of confusion and difficulty with language skills. She had become reclusive and disengaged from her previous social networks, and no longer attended any community activities or events. During seasonal changes she gradually became disorientated, leading to periods of wandering and becoming lost. She was also collecting and hoarding items of rubbish. The patient's son and daughter were rarely able to visit due to work and family commitments. However, her son recognised the patient's deterioration and provided financial support for her care.

Clinical findings: In September 2013, a local physician made a presumptive diagnosis of Alzheimer's disease based on her presenting symptoms. A Mini Mental State Examination (MMSE) score of 11 was determined. The clinical features were typical for Alzheimer's disease as the patient presented with impaired communication, disorientation, confusion, poor judgement, behavioral changes, and difficulty speaking.^[22]^ Depression was considered a differential diagnosis, however, apathy and depression are also known early neuropsychiatric symptoms of Alzheimer's disease.^[22]^ The patient had no significant past medical or psychiatric history and was not on any regular medications, traditional Chinese medications or supplements. Her physician recommended a medication for Alzheimer's disease, but the patient declined it. Her diet was poor consisting mainly of sugar buns and tea; consequently, she was underweight. Support services previously arranged by the patient's son had not lasted as the patient believed she was independent enough to function at home without help.

Timeline: The patient's timeline is displayed in Table 1.

**Table 1:**
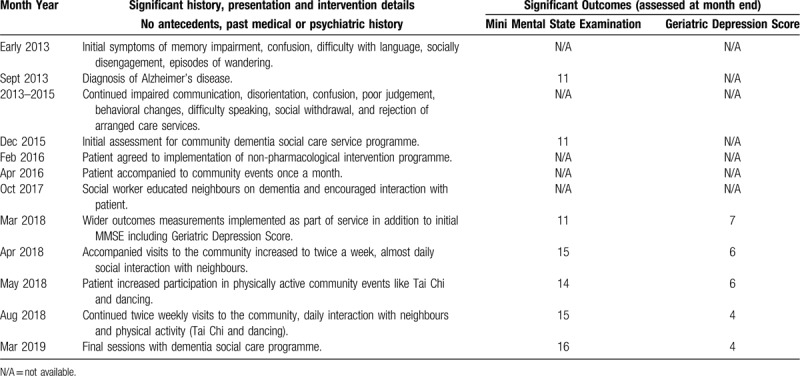
Patient timeline with significant outcomes displayed.

Diagnostic assessment: Dementia guidelines recommend exclusion of other causes of cognitive decline with blood and urine tests, followed by referral to a memory clinic.^[23]^ However, due to lack of clinical and financial resources, diagnosis was made based on examination and clinical history which is the current pragmatic approach to diagnosis in this patient's community.^[2,3]^ The local physician provided the presumptive diagnosis of Alzheimer's disease to the patient and family based on the patient's symptoms of impaired communication, disorientation, confusion, poor judgement, and behavioral changes.^[22]^ Depression was considered a differential diagnosis, however, apathy and depression are also known early neuropsychiatric symptoms of Alzheimer's disease.^[22]^ This diagnostic information was then provided to our social care manager. Assessment of the patient's scores in March 2018 were as follows: MMSE of 11,^[24–26]^ Clinical Dementia Rating Scale of 2 indicating moderate cognitive impairment,^[27,28]^ Global Deterioration Scale of 5 indicating moderately severe cognitive decline,^[29]^ Geriatric Depression Scale score of 7,^[30,31]^ and a Barthel Index of Activities of Daily Living score of 100.^[32,33]^

Therapeutic intervention: Our multicomponent dementia social care program, implemented by a social care manager and a social care worker with additional training in dementia, included:

- An in-depth needs assessment leading to a focused, practical care plan
- Non-pharmacological interventions to manage or better cope with symptoms of dementia and functional decline
- Community-based social activities targeted at reducing social isolation and re-integration of the patient into the community
- Dementia education and counselling for carers and surrounding communities to improve understanding, decrease stigma and assist with socialization.

The initial plan was for 3 h of non-pharmacological interventions delivered once a week. The patient was reluctant to start the program but after 2 months of regular visits from the social care worker, the patient agreed to start home-based sessions. Interventions offered included validation therapy,^[34]^ music therapy,^[8,9,12]^ art therapy,^[35]^ reminiscence therapy,^[8]^ talking therapy,^[36]^ reality orientation,^[8,34]^ cognitive training,^[8]^ smell therapy,^[9]^ food therapy,^[37]^ sensory stimulation,^[9]^ garden therapy,^[12]^ and physiotherapy.^[9]^ From March 2018, selection of the personalized non-pharmacological interventions was supported by a structured cognitive intervention pathway developed with input from an academic partner and implemented by the care team as a decision-support tool within the community delivery model. The structured cognitive intervention pathway aligns non-pharmacological interventions with relevant stages and symptoms of dementia based on the available published evidence for each intervention. Following assessment of the patient's needs, interests, preferences, symptoms, and stage of dementia, the pathway facilitated appropriate selection of non-pharmacological interventions. At each intervention episode, the Menorah Park Scale Engagement scale for Dementia was used to determine the patient's level of constructive, other or no engagement from activities.^[38]^ Mood was measured utilizing a 3-point Likert Scale of positive, neutral, and negative mood.^[39,40]^Table 2 shows the details of the interventions, episodes, and associated overall engagement and mood scores.

**Table 2:**
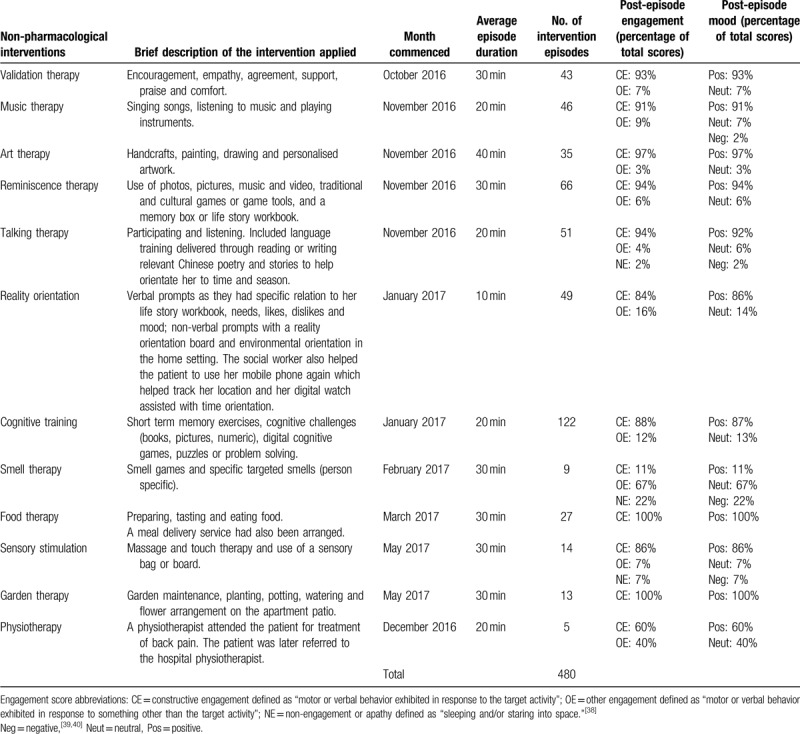
Non-pharmacological interventions delivered to the patient between October 2016 and March 2019 listed chronologically from month of initiation including number of intervention episodes and post-episode engagement and mood.

In parallel with one-to-one intervention sessions, the care team liaised with local community members regarding re-integration and inclusion in the community. From April 2016, our social worker accompanied the patient once a month to local cultural community events including music and poetry, Dragon Boat, Moon Cake, and Chinese New Year events. In October 2017 our social worker approached the patient's neighbors to educate them on Alzheimer's disease, the lived experience of a person with dementia and to encourage them to increase their interactions with the patient. After a long period of mostly home-based sessions, from April 2018, the patient was encouraged to socialize more frequently within the community and with her neighbors. The social worker accompanied the patient on visits to the community twice a week and the patient socialized almost daily with her neighbors. From May 2018, the patient participated in more active community events which included visiting Tiananmen Square, and physical activities including Tai Chi and light dancing.

**Figure 1:**
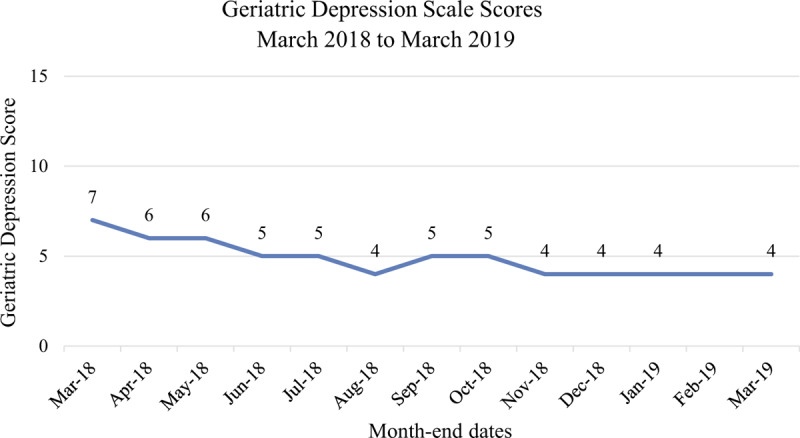
A graph of the patient's Geriatric Depression Scale Scores March 2018 to March 2019. Geriatric Depression Score-15: 0 to 5 normal; more than 5 indicates depression.^[30,31]^ (February 2019: no assessment due to Chinese holidays).

**Figure 2:**
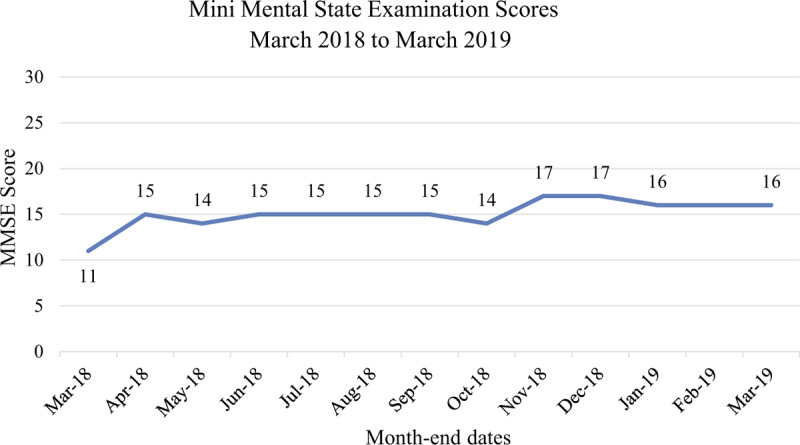
A graph of the patient's Mini Mental State Examination Scores March 2018 to March 2019. MMSE is scores out of 30 indicating levels of cognitive impairment severity as follows: 0 to 9 severe; 10 to 18 moderate; 19 to 23 mild.^[24–26]^ (February 2019: no assessment due to Chinese holidays).

Follow-up and outcomes: After the increase in social engagement and active community participation, an improvement in the patient's depression and MMSE scores was noted. The patient's Geriatric Depression Scale score (Fig. 1) progressively improved over the months from March to August 2018 when active social community participation increased. The largest jump in the patient's MMSE score (Fig. 2) was between March and April 2018 when the patient's social participation in the community and time spent outdoors increased.^[20,41]^ The Clinical Dementia Rating Scale of 2 (moderate cognitive impairment), Global Deterioration scale of 5 (moderately severe cognitive decline) and Barthel Index score of 100 remained unchanged over the 12 months.

Interventions that were associated with highest levels of constructive engagement and more positive mood included three of the patient's former hobbies which have been restored: music, art, and gardening. Other domains of improvement included language skills and communication, reduced hoarding, and weight gain (3.6 kg). Increased neighborhood awareness of the patient's condition has enhanced her safety during periods of confusion or wandering. There were no adverse effects reported.

In March 2019, the family employed a domestic worker to live in with the patient to help with cooking and accompany her to community activities. The social care team provided the domestic worker with 6 h of training on person-centred dementia care including some non-pharmacological activities. The patient's son has provided positive feedback about the service. The social care worker still maintains telephonic contact with the patient once a month.

## 3 Discussion

The community-based, multicomponent dementia social care program described in this case report has enabled a socially isolated patient living at home with symptoms of Alzheimer's disease including depression, to re-integrate into her community and return to active participation in cultural activities and former hobbies. Reduction in her social isolation through increased frequency of active community participation was associated with improvement in her mood and prevention of cognitive decline despite her refusal to take any medications for dementia. The increase in the MMSE was noted when the patient's social participation in the community and time spent outdoors increased.^[20,41]^ Time spent outdoors has also been associated with improvement in mood in people with moderate and severe dementia.^[41]^ The MMSE score remained between 14 and 17 over the following 12 months with no decline observed. It can be expected that the MMSE score would drop between 1.8 and 6.7 points annually for people with dementia.^[42,43]^ We acknowledge that the MMSE is intended for use as a screening tool and has practice effects when used repeatedly, however, it is still used in clinical settings to measures cognitive change over time, takes only 5 to 10 min and can be more readily administered by allied health professionals,^[44]^ compared to, for example, the Alzheimer's Disease Assessment Scale—Cognitive section (ADAS-Cog) which takes 40 min.^[45]^ While the ADAS-Cog would be optimal for future planned research, duration, and practical implementation of assessments within services need to be scalable in a large population.

Multimodal non-pharmacological interventions have demonstrated improvement in dementia symptoms,^[17]^ improvement in cognition,^[14,18,19]^ delay in cognitive decline,^[14,19]^ improvement in social behavior and social outcomes,^[14,16,17]^ and improvement in mood and behavioral outcomes for people living with dementia.^[14,15,16,18]^ However, those interventions specifically noted to be delivered in the community were based on short-term interventions without sustained improvement.^[18]^ Furthermore, there is little guidance available on how to implement a community model of non-pharmacological care in a person's home and select non-pharmacological interventions in a rational and systematic way. While the staff applied all the knowledge available, and further resources such as the structured cognitive intervention pathway were developed to facilitate intervention selection and monitoring, we acknowledge that, with a very limited home-care system in China,^[2]^ development and further research of a structured, multicomponent model of home-focused care for people with dementia within the community is crucial. Programs designed to facilitate maintaining independence, quality of life and integration with the community, that can be delivered by a range of social and allied health care professionals and informal carers could help reduce the burden on the formal health care sector. The fundamental factors of education and reducing social isolation are essential to successful implementation of care for people with dementia living alone at home.

## Acknowledgments

The authors would like to thank Charles Young for his editorial oversight and input.

## Author contributions

**Conceptualization:** Mark Mclean Carter, Angelina Wei, Xinlei Li.

**Data curation:** Mark Mclean Carter, Angelina Wei, Xinlei Li.

**Formal analysis:** Mark McLean Carter, Angelina Wei, Zara Quail.

**Writing – original draft:** Zara Quail, Mark McLean Carter.

**Writing – review & editing:** Zara Quail, Mark McLean Carter.
